# Lack of RNase L Attenuates Macrophage Functions

**DOI:** 10.1371/journal.pone.0081269

**Published:** 2013-12-04

**Authors:** Xin Yi, Chun Zeng, Hongli Liu, Xiaoli Chen, Ping Zhang, Boo Seok Yun, Ge Jin, Aimin Zhou

**Affiliations:** 1 Clinical Chemistry Program, Department of Chemistry, Cleveland State University, Cleveland, Ohio, United States of America; 2 Center for Gene Regulation in Health and Diseases, Cleveland State University, Cleveland, Ohio, United States of America; 3 Department of Cancer Biology, Lerner Research Institute, Cleveland Clinic, Cleveland, Ohio, United States of America; 4 Department of Biological Sciences, Case Western Reserve University School of Dental Medicine, Cleveland, Ohio, United States of America; 5 Central Laboratory, the Eighth Hospital of Xi'an, Xi'an, China; 6 Department of Pathology, the Second Affiliated Hospital of Medical School, Xi'an Jiaotong University, Xi'an, China; 7 Department of Pathology, Wanjing Hospital, China Academy of Chinese Medical Sciences, Beijing, China; The University of Texas Health Science Center at San Antonio, United States of America

## Abstract

**Background:**

Macrophages are one of the major cell types in innate immunity against microbial infection. It is believed that the expression of proinflammatory genes such as tumor necrosis factor-α (TNF-α), interleukin (IL)-1β, IL–6, and cyclooxygenase-2 (Cox-2) by macrophages is also crucial for activation of both innate and adaptive immunities. RNase L is an interferon (IFN) inducible enzyme which is highly expressed in macrophages. It has been demonstrated that RNase L regulates the expression of certain inflammatory genes. However, its role in macrophage function is largely unknown.

**Methodology:**

Bone marrow-derived macrophages (BMMs) were generated from RNase L^+/+^and ^−/−^ mice. The migration of BMMs was analyzed by using Transwell migration assays. Endocytosis and phagocytosis of macrophages were assessed by using fluorescein isothiocyanate (FITC)-Dextran 40,000 and FITC-*E. coli* bacteria, respectively. The expression of inflammatory genes was determined by Western Blot and ELISA. The promoter activity of Cox-2 was measured by luciferase reporter assays.

**Conclusions/Findings:**

Lack of RNase L significantly decreased the migration of BMMs induced by M-CSF, but at a less extent by GM-CSF and chemokine C-C motif ligand-2 (CCL2). Interestingly, RNase L deficient BMMs showed a significant reduction of endocytic activity to FITC-Dextran 40,000, but no any obvious effect on their phagocytic activity to FITC-bacteria under the same condition. RNase L impacts the expression of certain genes related to cell migration and inflammation such as transforming growth factor (TGF)-β, IL-1β, IL-10, CCL2 and Cox-2. Furthermore, the functional analysis of the Cox-2 promoter revealed that RNase L regulated the expression of Cox-2 in macrophages at its transcriptional level. Taken together, our findings provide direct evidence showing that RNase L contributes to innate immunity through regulating macrophage functions.

## Introduction

Macrophages function in both innate and acquired immune responses. In tissues, macrophages stand guarding against pathogen invading and are able to immediately defend and migrate to the sites of infection as well as present an antigen to the cells of the adaptive immune system. As a key player in the first line of defense, macrophages are distributed in all organs, tissues and fluids throughout the body. Upon triggered by a range of stimuli including damaged cells, pathogens and cytokines, macrophages are attracted by chemical substances through chemotaxis to a damaged or infected site. At the infected tissue sites, macrophages engulf and digest cellular debris and pathogens, subsequently activate lymphocytes or other immune cells in adaptive immunity [1], [2]. Macrophages show a great diversity of phenotypes and functions as a result of what they adapt to the microenvironment, where macrophages are exposed to particular tissues, cell types, and physiological states. Thus, macrophages from different location in the body vary in their maturation, function, and metabolism as evidenced by their differential responses to stimulation and displaying of different surface markers, even the distinct capability of phagocytosis: a hallmark of macrophage activity. For example skin-associated macrophages and Langerhans cells are poorly phagocytic. Furthermore, the expression of receptors, oxidative burst and cytokine production are markedly variable in macrophage subtypes as well [3]–[5].

A number of gene products have been found to mediate macrophage functions. In addition to a pathogen and altered cells, cytokines such as IFN-γ and TNF-α are able to activate macrophages. The recruitment of macrophages to the inflammatory site is a complex process involving their adhesion to endothelial cells, traverse of the perivascular connective tissue, and migration driven by a chemotactic gradient. CCL2, also known as monocyte chemoattractant protein 1 (MCP-1), is a member of the cytokine/chemokine superfamily promoting the migration of monocytes and macrophages to the sites of inflammation [6]. It has been shown that mice deficient CCL2 decrease recruitment of macrophages in response to infection [7], [8]. IL-10 is able to facilitate macrophage migration through its inhibitory effect on macrophage migration inhibitory factor (MIF) [9], [10]. Growth factors such as TGF-β and M-CSF also contribute to macrophage recruitment at the inflammatory sites and tumor tissues [11], [12]). Interestingly, it has been reported that macrophages secret TGF-β, which in turn stimulates their migration through inducing the expression of CCL2 mediated by RhoA [13]. Cox-2, a key regulator in inflammation, is also involved in macrophage migration and infiltration. A Cox-2 inhibitor is found to completely inhibit macrophage migration [14]. In the brain of patients with Alzheimer's disease, only Cox-2 positive macrophages infiltrate into perivascular spaces and neuropil [15].

RNase L is one of the key enzymes in the 2-5A system of IFN action against viral infection and cellular proliferation [16], [17]. The 2-5A system consists of two enzymes: 2-5A synthetase and RNase L. IFNs induce the expression of a family of 2-5A synthetase genes (OAS). Activation of 2-5A synthetases requires double-stranded RNA (dsRNA), which is frequently produced during viral infection. After activation by dsRNA, 2-5A synthetases convert ATP molecules to pyrophosphate (ppi) and a series of unique, 5′-phosphorylated, 2′-5′ linked oligoadenylates known as 2-5A with the general formula of ppp(A2′p5′)nA (*n*≥2). 2-5A activates RNase L resulting in degradation of single-stranded viral and cellular RNAs, leading to inhibition of the replication of certain viruses and cell proliferation [18]–[20]. RNase L null mice show enlarged thymus glands and increased T-cell numbers at the early age, suggesting that RNase L may be involved in T-cell development, which likely results from reduced cell apoptosis. Furthermore, it has been demonstrated that overexpression of RNase L in the cells enhances cell apoptosis, whereas dominant negative RNase L suppresses cell apoptosis [21]. These observations implicate that RNase L may play an important role in the immune system. Indeed, studies have revealed that skin allograft rejection is suppressed in mice lacking RNase L, suggesting the involvement of RNase L in T-cell immunity, particularly CD4^+^ T-cell mediated immunity [22]. In addition, alphavirus-based DNA vaccination against a non-mutated tumor-associated self-antigen (tyrosinase-related protein-1, TRP-1) is severely impaired in RNase L null mice, indicating that RNase L plays an important role in the host immune system against cancer [23].

RNase L is present at basal levels in most mammalian cells. Tissue distribution analysis has revealed that RNase L is highly expressed in the spleen, thymus, and most of immune cells such as T, B cells and macrophages. However, the physiological role of RNase L in the immune system is largely unknown. In the present study, we demonstrate that RNase L contributes to macrophage functions by using BMMs from RNase L deficient and wild type mice. Furthermore, RNase L regulates the expression of Cox-2, and certain cytokines and chemokines in macrophages, which in turn modulates macrophage functions. Taken together, our findings suggest a novel role of RNase L in innate immunity.

## Results

### RNase L deficiency reduces macrophage migration

**Figure 1:**
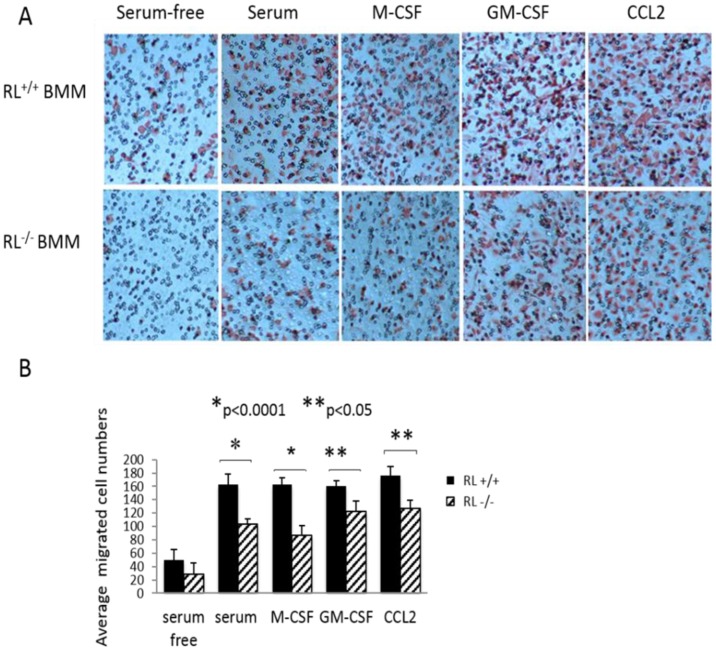
Effect of RNase L on macrophage migration. (A) RNase L deficient and wild type BMMs were seeded to the upper chambers of 8 µm Transwells (black) precoated with 20 µg/ml fibronetin for overnight at 4°C. In the lower chambers, serum-free DMEM, serum-free DMEM with 10% FBS or 100 ng/ml of M-CSF or GM-CSF or CCL2 were added respectively, and the cells were allowed to migrate at 37°C overnight. The cells on the filter side of the up chambers were cleaned with a cotton swab and the migrated cells (red) were fixed by 10% formalin for 5 min, followed by staining with eosin. The unstained grey circles were the pores of the filter. The experiment was repeated three times in duplicate for each treatment. The photos were from one of the three experiments. (B) The cell numbers were averaged from five fields per transwell in duplicate and present as Mean ± SD. *p<0.0001, **p<0.05.

RNase L is highly expressed in macrophages [24]. To determine the effect of RNase L on macrophage functions, we first examined the migration of BMMs with or without RNase L under the influence of different chemotrafficking reagents. In this experiment, BMMs differentiated from the bone marrow cells of RNase L ^+/+^ and ^−/−^ mice in serum-free medium were seeded in the upper chambers of the transwells with a pore size of 8 µm, a commonly used tool to analyze cell migration. The media in the lower chambers were supplemented with or without 10% fresh fetal bovine serum, 100 ng/ml of M-CSF, GM-CSF and CCL2, respectively. The cells on the upper layer of the membrane migrated through the micropores on the membrane while sensing chemoattractants in the lower chamber. After termination of the experiment, the migrated cells were stained with eosin. As shown in Figure 1A, lack of RNase L significantly reduced the migration of BMMs, particularly in the presence of fresh serum, M-CSF, GM-CSF, and CCL2. Quantitative analysis revealed that lack of RNase L reduced 19.4%, 36%, 47%, 24%, and 28% of the population of migrated cells under the condition without or with serum, M-CSF, GM-CSF, and CCL2, respectively (Figure 1B). Obviously, RNase L mostly impacted the M-CSF-induced macrophage migration although it also contributes to the chemotrafficking capability of other factors.

### RNase L contributes to macrophage endocytosis, but not phagocytosis

**Figure 2:**
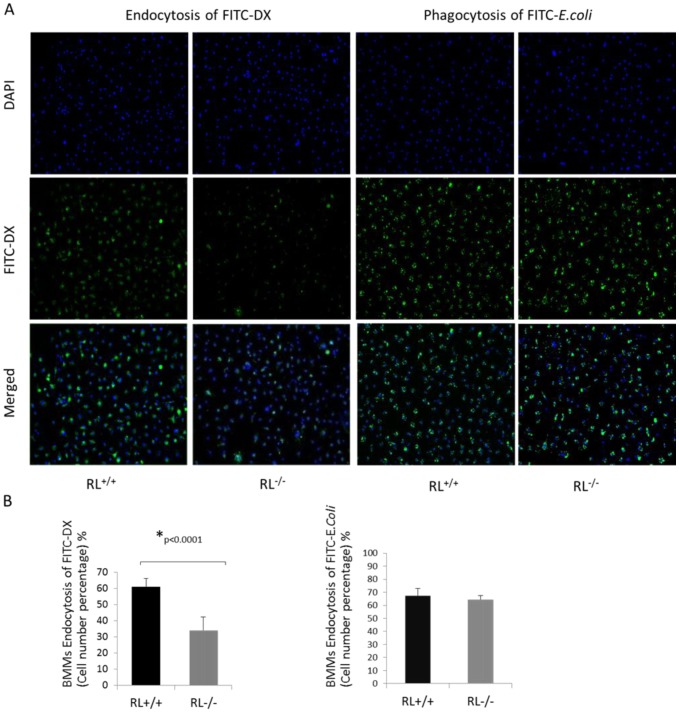
Role of RNase L in macrophage endocytosis and phagocytosis. (A) RNase L deficient and wild type BMMs were activated with LPS (0.5 µg/ml) for 48 hours, followed by incubating with FITC-Dextran 40,000 (1 µg/ml) or FITC-*E*. *coli* (bacterial to macrophage ratio = 50∶1) at 37°C for 1 h. After incubation, the cells were fixed with 4% paraformalhyde for 10 min and washed thoroughly with ice cold phosphate-buffered saline (PBS). Extracellular fluorescence was quenched by trypan blue. The cells with fluorescence were photographed under a fluorescent microscope. The photos are shown from one of five experiments. (B) The fluorescent cell numbers were averaged from eight fields per well and present as Mean ± SD, *p<0.0001.

The main function of macrophages in cell physiology and pathology is their capability of endocytosis and phagocytosis. To determine the effect of RNase L on macrophage endocytosis and phagocytosis, RNase L^+/+^ and ^−/−^ BMMs were incubated with either FITC-Dextran 40,000 or FITC-*E. coli* after the activation by lipopolysaccharine (LPS). FITC-Dextran is commonly used as a fluorescent probe to study cell permeability or endocytosis and FITC-*E. coli* is employed to analyze macrophage phagocytosis [25]. Fluorescence of engulfed FITC-Dextran or -E. *coli* was observed and analyzed under a microscope after washing to remove the rest of them in the media and quenching the extracellular signals. As shown in Figure 2A, deficiency of RNase L significantly reduced the capability of BMMs to endocytosize FITC-Dextran 40,000, but had nearly no effect on their phagocytotic function in engulfing bacteria. The ratio of the average number of the cells with fluorescence to the total cells from 8 fields was 61.1±5.0% in RNase L^+/+^ BMMs compared to 34.0±8.3% in RNase L^−/−^ BMMs in the assay with FITC-Dextran 40,000. However, the ratio of the fluorescent cells to the total cells after incubation with FITC-*E.coli* was 67.4±5.4% and 64.5±3.0% in RNase L^+/+^ and ^−/−^ BMMs respectively, under the same condition (Figure 2B). The result indicates that lack of RNase L in macrophages significantly reduces their endocytotic, but not phagocytotic function. However, the molecular mechanism remains largely unknown.

### RNase L regulates the cytokine production in macrophages

**Figure 3:**
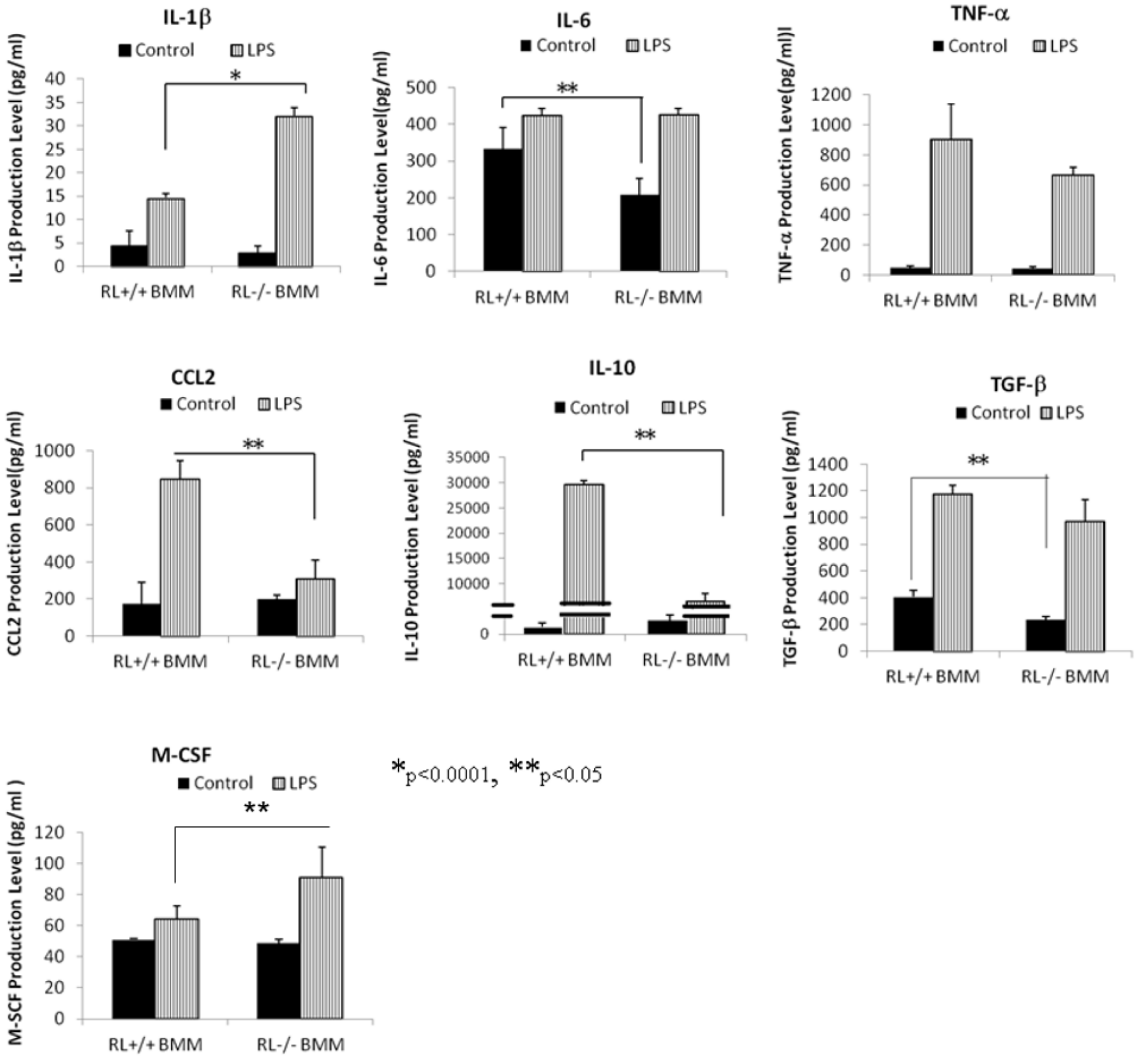
RNase L regulates the expression of cytokines and chemokines. RNase L deficient and wild type BMMs were treated with 1 µg/ml of LPS for 14 h. The secretory level of certain cytokines and chemokines in the media was measured by using an ELISA kit for each of the analyzers. Experiments were performed two times in triplicates. Data are presented as Mean ±SD. *p<0.0001, **p<0.05.

It has been well known that macrophages are able to secret a large number of cytokines, chemokines and growth factors under stimulation, which in turn regulate their own functions and promote adaptive immunity [26]. Since our results showed that RNase L was involved in macrophage migration and endocytosis, we were intrigued to determine if RNase L regulates the production of these factors contributing to the function of macrophages. RNase L ^+/+^ and ^−/−^ BMMs were treated with LPS and the secreted products of TNF-α, CCL-2, IL-1β, IL-6, IL-10 and TGF-β, which are believed to be associated with cell migration and macrophage functions, in the media were analyzed by using ELISA. As shown in Figure 3, RNase L is necessary for the efficient induction of CCL-2 and IL-10, and lack of RNase L reduced 63% and 78% of the secretory level of CCL-2 and IL-10, respectively, under stimulation with LPS. However, RNase L only has a modest effect on the production of TNF-α and TGF-β. Although the basal level of IL-6 in RNase L^+/+^ BMMs is significantly higher (25%) than that in RNase L^−/−^ BMMs, RNase L seemed not to contribute to the induction of IL-6 at all in the cells by LPS. Interestingly, RNase L apparently inhibited the production of IL-1β and M-CSF after the cells were treated with LPS. The secretory level of IL-1β and M-CSF was 2.3- and 1.3-folds higher respectively in the medium of BMMs deficient RNase L than that in the medium of wild type BMMs. To confirm the results, we generated an RNase L deficient Raw 264.7 cell line, a mouse macrophage cell line, in which RNase L was knocked down by using RNase L hairpin RNA (ShRNA) lentiviral particles (Figure S1). RNase L knocked down Raw 264.7 cells were treated with LPS and the level of these factors described above in the media was determined by ELISA. As expected, the results were similar to that obtained in BMMs (Figure S2).

### RNase L mediates the Cox-2 expression at its transcriptional level

**Figure 4:**
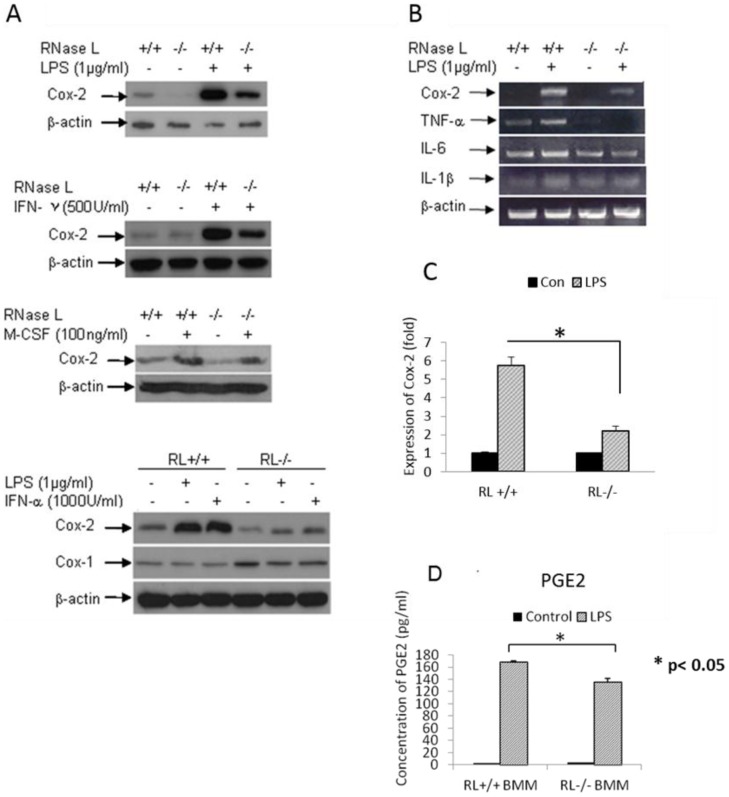
RNase L regulates the expression of Cox-2. RNase L deficient and wild type BMMs were treated with 1 µg/ml of LPS, 1,000 units/ml of IFN-α, 500 units/ml of IFN-γ, and 100 ng/ml of M-CSF for 14 h. The expression of Cox-2 was determined by Western blot analysis using a monolocnal antibody against mouse Cox-2 (A). RNase L wild type and deficient MEFs were treated with 1 µg/ml of LPS for 14 h. Total RNAs were isolated by using the Trizol Reagent (Invitrogen, CA). The expression of Cox-2, TNF-α, IL-6 and IL-1β was determined by RT-PCR (B). The expression of Cox-2 was measured by real-time PCR (C), which was performed twice in triplicate; the fold change from one of the experiments is present as Mean ±SD, *p<0.05. The production of PGE2 in the media culturing the two types of BMMs treated with LPS as described above was analyzed by ELISA (D). Experiments were performed twice in triplicates. Data are presented as Mean ±SD, *p<0.05.

To determine if RNase L is involved in regulating the expression of other proinflammatory genes, we examined the expression of Cox-2, which catalyzes the production of prostaglandins and other eicosanoids, to promote inflammation and is associated with macrophage function [14], [15]. In the experiment, RNase L^+/+^ and ^−/−^ BMMs were treated with LPS and other stimuli, and the expression of Cox-2 was determined by Western blot, RT-PCR and qPCR. As shown in the Figure 4A, the expression of Cox-2 was significantly induced in RNase L ^+/+^ BMMs after the cells were treated with LPS, M-CSF, IFN-α and -γ. However, the induction of Cox-2 was remarkably attenuated in RNase L^−/−^ BMMs, suggesting that RNase L impacts the expression of Cox-2 in the cells induced by LPS. Similar results were also obtained by using mouse embryonic fibroblasts (MEF) after treated with LPS and the samples were analyzed by RT-PCR (Figure 4B). qPCR analysis revealed that the expressing level of Cox-2 in RNase L ^+/+^ BMMs after treatment with LPS was about 3-fold higher than that in RNase L^−/−^ BMMs (Figure 4C). Overall, our results demonstrate that RNase L is involved in the induction of Cox-2 by LPS. To confirm that the increased expression level of Cox-2 results in enhancement of the Cox-2 enzymatic activity in the cells, the production of PGE2 was measured by ELISA. As expected, the production of PGE2 was about 20% higher in the medium of RNase L^+/+^ BMM in compared to that in the medium of RNase L^−/−^ BMM after the cells were treated with LPS (Figure 4D).

**Figure 5:**
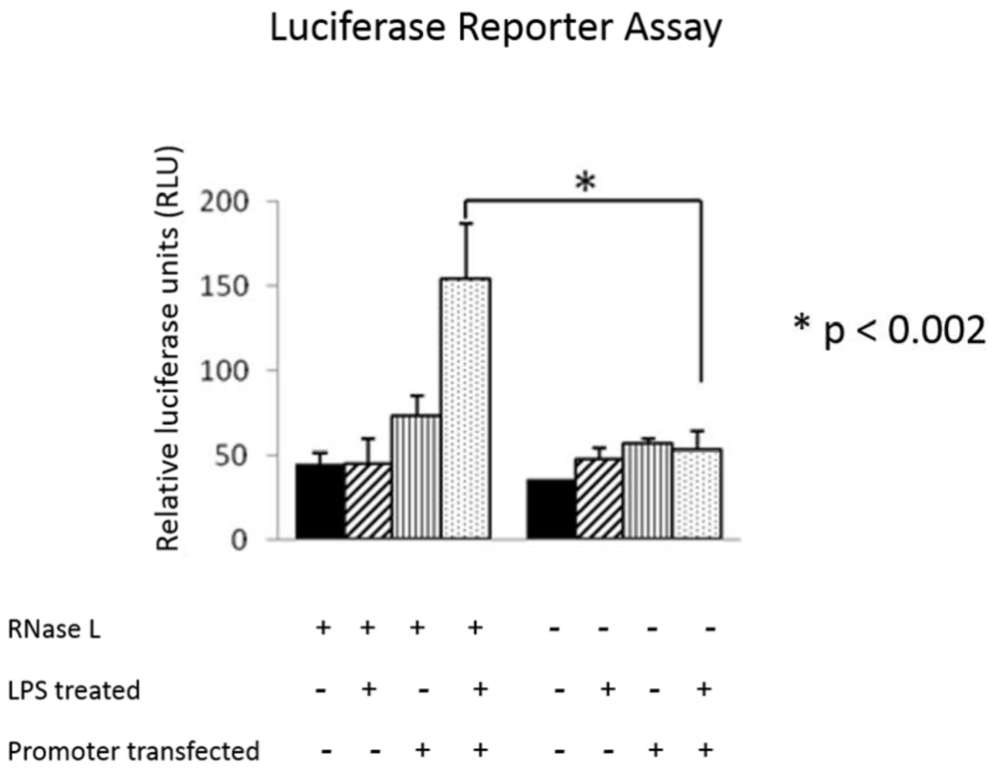
Promoter activity analysis of Cox-2. RNase L deficient and wild type MEFs were transfected with the Cox-2 promoter (−860/+127)-pGL3 luciferase reporter construct (20 µg) together with a plasmid containing a β-galactosidase gene (1 µg) by using lipofectamine 2000 (Invitrogen, CA) according to the manufacture's instruction. After transfection, the cells were allowed to recover for 24 h and subsequently treated with or without LPS (1 µg/ml) for 14 h. The activity of luciferase and β-galactosidase was assayed as described in Materials and Methods. The experiments were performed twice in triplicates and the result are present as Mean ± SD. *p<0.002.

It has been well known that RNase L plays an important role in RNA turnover after activation by 2-5A during viral infection, resulting in regulating the production of certain genes [27]. To determine the level by which RNase L regulates the expression of Cox-2, the promoter activity of Cox-2 was analyzed in RNase L^+/+^ and ^−/−^ cells. RNase L ^+/+^ and ^−/−^ MEFs were transfected with a reporter construct containing a luciferase gene under the control of Cox-2 (−860/+127) promoter [28] and the cells were then treated with or without LPS (1 µg/ml) for 14 h. The luciferase activity was measured by using a luciferase activity assay kit (Promega, MO) according to the manufacture's instruction. The β-galactosidase gene was co-transfected and served as an internal control for transfection efficiency. Clearly, the promoter activity of Cox-2 was remarkably higher in the RNase L^+/+^ cells than that in the RNase L^−/−^ cells, suggesting that RNase L may regulate the expression of Cox-2 at its transcriptional level (Figure 5).

## Discussion

The role of RNase L in IFN functions against viral infection and cell proliferation has been well established. In recent years, RNase L is found to be involved in apoptosis and autophagy as well [18], [29]. In this study, we demonstrate that RNase L contributes to macrophage functions including endocytosis, migration, and cytokine production, which are crucial in innate immunity. Although it has been reported that RNase L is associated with innate immunity through regulating the antiviral immune response [30], this is the first study showing that RNase L impacts innate immunity by directly mediating the function of immune cells such as macrophages. The findings significantly expand our horizon of understanding the basic cellular functions and signaling pathways of RNase L.

Macrophage migration is one of the major characteristics of macrophage functions involved in several pathological situations including cancer, neurodegenerative disorders and chronic inflammation. It has been believed that macrophages can use either the amoeboid (protease-independent) or the mesenchymal (protease-dependent) migration mode depending on the environmental constraints to migrate to the site of infection and targeting spots [31]. Macrophages are recruited to specific sites by chemotaxis which directs cell migration towards chemoattractant gradients. The cell senses a gradient and moves by extending actin-rich protrusions towards the chemoattractant propelled by actin-myosin mediated contractions [32]. RNase L seems to play a critical role in the M-CSF induced cell migration and deficiency of RNase L in macrophages reduced about 50% of migrated cell population. M-CSF is a pleiotropic macrophage growth factor, which contributes to the differentiation, survival, proliferation and motility of mononuclear cells. M-CSF regulates the function of macrophages through activation of the tyrosine kinase receptor CSF-1R, leading to phosphorylation of downstream molecules and resulting in activation of several important signaling transduction pathways including PI-3K. PI3K signals primarily through Akt to activate Rac and inactivate Rho, to stimulate actin polymerization, cytoskeletal remodeling and cell adhesion, subsequently promote cell moving [33]. Although we have not determined if RNase L is involved in M-CSF-induced macrophage migration through impacting on the PI-3K pathway, our unpublished result indeed revealed that phosphorylation of Akt in RNase L deficient MEFs and primary hepatocytes was remarkably inhibited under stimulation with insulin, suggesting that blockage of the PI-3K pathway may be the cause to reduce the migrated population of RNase L deficient macrophages. The role of RNase L in CCL2 -and GM-CSF –induced macrophages remains to be elucidated.

Phagocytosis and endocytosis by which macrophages, dendritic cells and other myeloid phagocytes internalize diverse microbes and particulate targets are the vital mechanism of both innate and adaptive immunities. The mechanisms mediating the internalization of target particles are distinct, depending on a specific target and its location. A variety of different receptors on the surface of these phagocytes are able to sense and recognize their cognate ligands expressed on microorganisms and soluble matter, and subsequently ingest them by a phagocytic or endocytic manner [3], [5]. These processes are essential for macrophage antigen presentation to activate T-cells, and mostly promote the expression of proinflammtory cytokines and chemokines, which orchestrate local and systemic inflammatory responses, leading to the development of adaptive immunity. Interestingly, RNase L seems to be involved in only engulfing dextran, not E. coli bacteria by macrophages, suggesting that RNase L may contribute to the function of a specific receptor responsible for intake of certain types of particulates. Many factors are associated with efficacy of phagocytosis and endocytosis of macrophages, for example, the size and other physical properties, such as geometry and topography of a target molecule can influence its delivery into the cells [34]. However, receptors are believed to play a critical role in the phagocytic uptake of target particles. It has been known that FITC-Dextran is mainly taken up through the mannose receptor, a C-type lectin, into macrophages [30], [31]. Most recently, a study has revealed that the mannose receptor is required for the endocytic uptake of influenza virus into macrophages [35]. RNase L is a well-known antiviral protein mediating IFN functions *in vitro* and *in vivo*, and also found to be located in cytoskeleton although its role in the cell membrane remains largely unknown [36]. Our results suggest that RNase L may function as a component necessary for the entry of certain types of virus into a cell, a role similar to CD4 and chemokine co-receptors such as CXCR4 or CCR5 in HIV infection [37], which subsequently trigger the apoptotic cascade, leading to the clearance of virus although this hypothesis needs to be further demonstrated. In addition, mannose receptors mediate phagocytosis of a variety of bacterium strains such as *Candida albicans*, *Zymosan*, *Pneumocystis carinii*, *Klebsiella,* and *mycobacteria*, as well as other pathogens [38]–[43]. Thus, there was no obvious difference in phagocytosis of FITC-*E. coli* between RNase L+/+ and −/− macrophages does not exclude the possibility that RNase L may mediate the process for the cell uptake of other types of microorganisms.

It has been believed that RNase L regulates the production of several genes such as TNF-α, IL-1β, and IFN-β during viral and bacterial infection [30], [44]. In this study, we determined the secretory level of TNF-α, CCL-2, IL-1β, IL-6, IL-10, TGF-β, and M-CSF, which may be associated with cell migration and macrophage functions, and found that RNase L was necessary for the efficient expression of CCL2 and IL-10, and modestly promoted the induction of TNF-α and TGF-β in the presence of LPS. In contrast, RNase L seemed to inhibit production IL-1β and M-CSF induced by LPS. It is surprising that deficiency of RNase L resulted in a 5-fold reduction of the expression of IL-10, a cytokine with broad anti-inflammatory properties involved in the pathogenesis of various diseases [45], in macrophages after treatment with LPS. On the other hand, the expression level of Cox-2 is 3-fold higher in RNase L+/+ cells than that in RNase L −/− cells under the treatment with LPS and other stimuli. Our results showed that RNase L may play a dual role in immunity by regulating the expression of proinflammatory and anti-inflammatory genes under a specific microenvironment although the physiological significance of the event needs to be further investigated. The promoter analysis revealed that RNase L regulates the expression of Cox-2 at its transcriptional level, but how and what is the molecular mechanism are completely unknown. RNase L has 9 typical anykrin-repeats and a conserved kinase domain in its protein structure. Hereby, it is possible that RNase L may directly interact with or phosphorylate some transcriptional factors to regulate the expression of Cox-2 in the cells, and this hypothesis is under investigating in the laboratory. Taken together, our findings provide new insight into the contribution of RNase L to macrophage functions, and suggest a novel role of RNase L in immunity.

## Materials and Methods

### Cell Culture

BMMs were generated from the bone marrow cells of RNase L deficient and wild type C57BL/6 mice (a generous gift from Dr. Robert Silverman, Cleveland Clinic) by using a modification of a previously reported method [39]. Briefly, bone marrow cells were isolated from the femurs of mice by repeated flushing of the bone shaft with cold medium and cultured in RPMI 1640 medium (Cleveland Clinic, Cleveland, OH) supplemented with 10 ng/ml recombinant murine M-CSF (Shenandoah Biotechnology, Warwick, PA), 20% fetal bovine serum (VWR, Bridge Port, NJ), and 10% L929 cells-conditioned medium (LCM). Macrophages were ready after 7 days culture under the condition. To assess macrophage endocytosis and phagocytosis capability, macrophages were activated with LPS (0.5 µg/ml) for 48 h before the experiments. This study was carried out in strict accordance with the recommendations in the Guide for the Care and Use of Laboratory Animals of the National Institutes of Health. The protocol was approved by the Committee on the Ethics of Animal Experiments of Cleveland State University (Permit Number: 21111-ZHO-AS). All efforts were made to reduce suffering. Mice were under CO_2_ for 5 min and then dislocated their neck before isolation of bone marrow cells.

### Knocking down of RNase L in RAW264.7 cells

RAW264.7 cells were cultured in a 12-well plate to 50% confluent on the day of infection. A complete medium with polybrene (Santa Cruz Biotechnology, Dallas, TX) at a final concentration of 5 µg/ml was added in each well and the cells then were infected by directly adding mouse RNase L shRNA or empty lentiviral particles (Santa Cruz Biotechnology, Dallas, TX). After incubation overnight, the culture medium was replaced with a fresh complete medium. Clones were selected by culturing the infected cells in the medium containing puromiycin (10 µg/ml) and the expression of RNase L in the clones was analyzed by Western blot.

### Endocytosis and phagocytosis assay

Endocytosis and phagocytosis of macrophages were assessed by using FITC-Dextran 40,000 (Sigma, St. Louis, MO) and FITC-*E. coli* particles (Invitrogen, Grand Island, NY) respectively. The cells were washed twice with cold PBS after macrophages were activated with LPS (0.5 µg/ml) for 48 hours, and incubated in fresh RPMI 1640 medium (Cleveland Clinic, Cleveland, OH) containing 10% FBS and 25 mM Hepes with FITC-Dextran 40,000 (1 µg/ml) or FITC-*E*. *coli* (bacterial to macrophage ratio = 50∶1) at 37°C for 1 h. Negative control groups were incubated on ice. After incubation, the cells were fixed with 4% paraformalhyde for 10 min and fluorescence of extracellular particles was thoroughly washed out by using ice cold phosphate-buffered saline (PBS) and quenched with 1–2 ml 0.02% trypan blue for 3 min. A cold anti-fade reagent (Invitrogen, Grand Island, NY) was used as a mounting solution and slides were observed under a microscope.

### Macrophage migration assay

BMMs (2×10^5^) in the serum free medium were seeded to the upper chambers of 8 µm Transwells (ThinCert^TM^-24 Well) (VWR, Bridge Port, NJ) precoated with 20 µg/ml fibronetin for overnight at 4°C. In the lower chambers, serum-free DMEM, serum-free DMEM supplemented with 10% FBS or 100 ng/ml of M-CSF or GM-CSF or CCL2 (Shenandoah Biotechnology, Warwick, PA) were added respectively, and the cells were allowed to migrate at 37°C overnight. At the end of the assay, the filter side of the up chamber was cleaned with a cotton swab and the migrated cells were fixed by 10% formalin for 5 min and then stained with eosin. Five fields per transwell were photographed at 100× magnification under a microscope and the cells in each image were counted.

### Western blot analysis

After treatment, cells were washed twice with ice-cold PBS and collected with a scraper. The cytoplasmic extracts were prepared by suspension of the cell pellets in the NP-40 lysis buffer (10 mM Tris-HCl, pH8.0, 5 mM Mg(OAc)_2_, 90 mM KCl, 0.2 mM PMSF, 100 units/ml aprotinin, 10 µg/ml leupeptin and 2% NP-40). After centrifugation at 10,000×g in a microcentrifuge at 4°C for 10 min, the cell extracts (100 µg per sample) were fractionated on SDS-10% polyacrylamide gels and transferred to PVDF membranes (Millipore, Billerica, MA). The membranes were blocked with 5% nonfat milk in PBS containing 0.02% sodium azide and 0.2% (v/v) Tween 20, and incubated with different primary antibodies for 1 h at room temperature. The membranes were then washed with PBS containing 0.2% (v/v) Tween 20 and incubated with specific secondary antibodies conjugated with horseradish peroxidase (Cell Signaling, Billerica, MA) for 1 h at room temperature. After washing, the proteins were detected by a chemiluminescent method according to the manufacturer's specification (Pierce, Rockford, IL).

### RT-PCR and real time PCR

The cells were treated with 1 µg/ml of LPS for 14 h and the total RNAs were isolated by using the Trizol reagent (Invitrogen, Grand Island, NY). RT-PCR was performed by using the Superscript One-Step RT-PCR kit (Invitrogen, Grand Island, NY). The condition for cDNA synthesis is: 1 cycle at 55°C for 30 min, and 1 cycle at 94°C for 2 min. PCR amplification was performed by using the following condition: denaturing at 94°C for 15 sec, annealing at 55°C for 30 sec, and extension at 72°C for 1 min, for 25 cycles, then an additional cycle at 72°C for 8 min. The PCR primers are listed below. Cox-2 sense: 5′ GCA AAT CCT TGC TGT TCC AAT C 3′, antinsense: 5′ GGA GAA GGC TTC CCA GCT TTT G 3′; TNF-a sense: 5′ GAT CTC AAA GAC AAC CAA CTA GTG 3′, antisense: 5′ CTC CAG CTG GAA GAC TCC TCC CAG 3′; IL-6 sense: 5′ CAT GTT CTC TGG GAA ATC GTG G 3′, antisense: 5′ AAC GCA CTA GGT TTG CCG AGT A 3′; IL-1b sense: 5′ GGG ATG ATG ATG ATA ACC TG 3′, antisense: 5′ TTG TCG TTG CTT GGT TCT CCT 3′; β-actin sense: 5′TCA CCC ACA TG TGC CCA TCT ACG A3′, antisense: 5′ CAG CGC AAC CGC TCA TTG CCA ATG G3′. qPCR for Cox-2 was performed by using QuantiTect SYBR Green PCR kit (Qiagen, Valencia, CA) in a Thermal Cycler (Model: DNA Engine 2/Opticon2).

### Enzyme-linked immunosorbent assay (ELISA)

Cytokines and other factors in the media culturing macrophages were measured by ELISA with commercial available kits (eBioscience, San Diego, CA and R&D Systems, Minneapolis, MN). Briefly, RNase L deficient and wild type BMMs were treated with 1 µg/ml of LPS for 14 h, and the media were collected and stored in a −80°C freezer before analysis. For the assays, flat bottom 96-well ELISA plates were coated with a capture antibody at 4°C according to manufacturer's instruction. After overnight incubation at 4°C, the plates were washed three times and blocked with the blocking buffer provided in the kit, and then incubated with standards and cell culture medium samples for 2 h at room temperature. After washing the plates, a specific biotinylated antibody was added to each well and incubated for 1 h, followed by washing and 30 min incubation with avidin peroxidase. Then, substrates containing 3, 3′ 5, 5′-tetramethylbenzidine (TMB) and hydrogen peroxide were added, and the reaction was terminated by adding 50 µl of phosphoric acid after 30 min. Plates were read at 450 nm in a 96-well LD 400 C microplate reader (Beckman Coulter, Fullerton, CA).

### Luciferase reporter assay

The Cox-2 promoter (−860/+127)-pGL3 luciferase reporter construct (20 µg) provided by Dr. Narkunaraja Shanmugam (Division of Diabetes, Beckman Research Institute of the City of Hope) was transfected into RNase L^+/+^ and ^−/−^ MEFs together with a plasmid containing a β-galactosidase gene (1 µg) by using lipofectamine 2000 (Invitrogen, Grand Island, NY) according to the manufacture's instruction. After transfection, the cells were allowed to recover for 24 h and subsequently treated with or without LPS (1 µg/ml) for 14 h. The cells were washed twice with PBS and harvested for luciferase and β-galactosidase activity assays. Briefly, the cell extracts (20 µl/sample) were added to the wells of a 96-well microtiter plate (Dynstech, Inc., Mclean, VA) and mixed with 50 µl luciferase assay substrates. The results were determined in a luminometer (Perkin Elmer, Waltham, MA). To monitor β-galactosidase activity, the cell extracts were incubated with the assay buffer (200 mM sodium phosphate buffer, pH 7.3, 2 mM MgCl2, 100 mM β-mercaptoethanol) containing the substrate O-nitrophenyl-β-D-galactopyranoside (ONPG) (Sigma, St. Louis, MO) at 37°C until a yellow color appeared, and the reaction was terminated by addition of sodium carbonate. The absorbance at 420 nm was measured by spectrophotometry.

## Supporting Information

Figure S1 **Knocking down of RNase L in mouse macrophages**. RAW264.7 cells were infected by mouse RNase L shRNA or empty lentiviral particles in the medium containing 5 µg/ml of polybrene. Clones were selected by culturing the infected cells in the medium containing puromiycin (10 µg/ml) and the expression of RNase L in the clones was analyzed by Western blot using a poly clonal antibody to mouse RNase L. β-actin was used to normalize the protein loading.
(TIF)

Figure S2 **RNase L regulates the expression of cytokines and chemokines in macrophages.** RNase L knocking down (Clone 106) and wild type Raw264.7 cells were treated with 1 µg/ml of LPS for 14 h. The secretory level of certain cytokines and chemokines in the media was measured by using an ELISA kit for each of the analyzers. Experiments were performed two times in triplicates. Data are presented as mean ±SD. *p<0.001, **p<0.05.
(TIF)
