# Supplementary figures and images for: Lack of RNase L Attenuates Macrophage Functions
Source: PLoS One. 2013 Dec 4;8(12):e81269. doi: 10.1371/journal.pone.0081269 (PMC3852499; doi:10.1371/journal.pone.0081269)

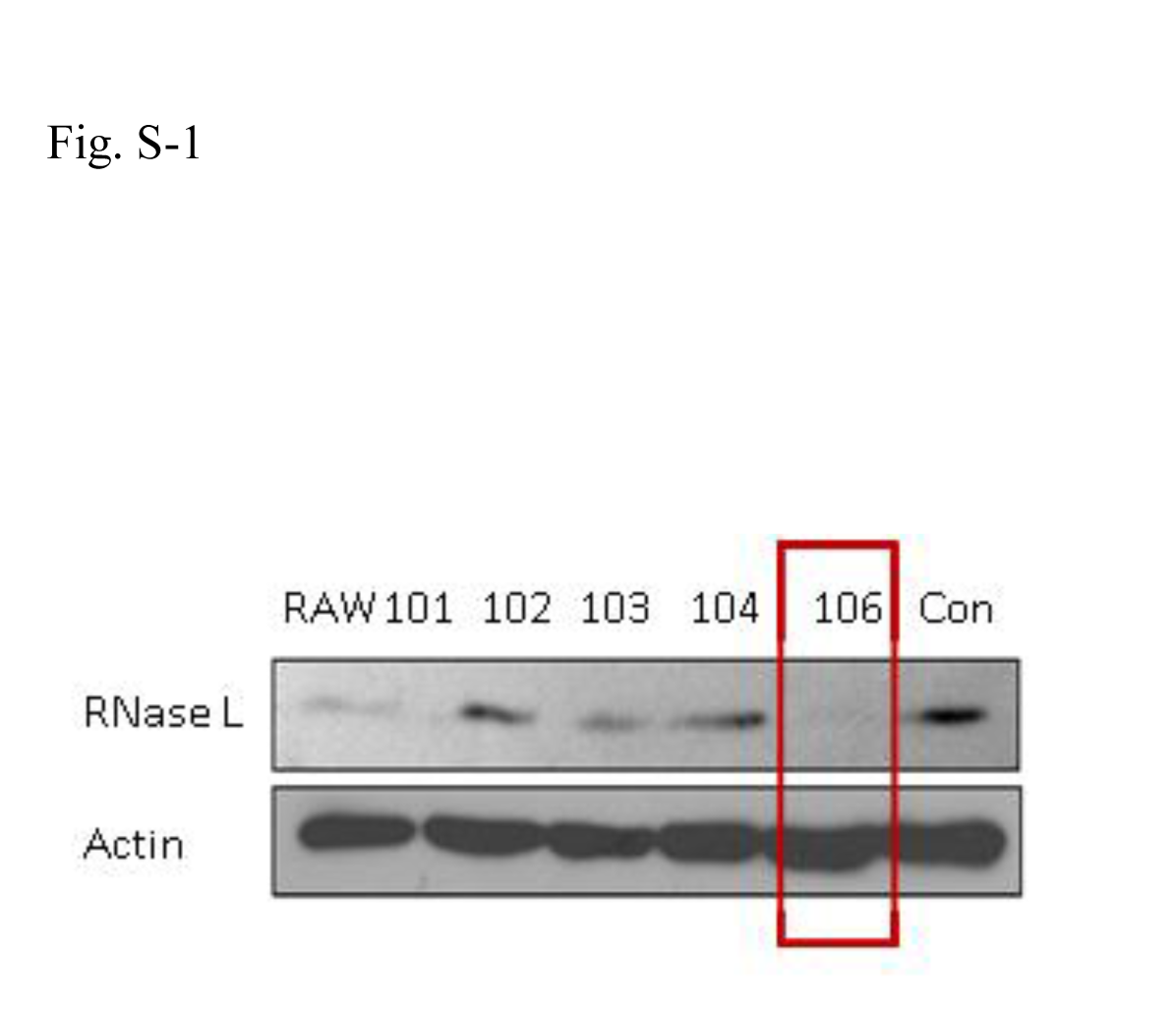

Supplement: Figure S1 — Knocking down of RNase L in mouse macrophages. RAW264.7 cells were infected by mouse RNase L shRNA or empty lentiviral particles in the medium containing 5 µg/ml of polybrene. Clones were selected by culturing the infected cells in the medium containing puromiycin (10 µg/ml) and the expression of RNase L in the clones was analyzed by Western blot using a poly clonal antibody to mouse RNase L. β-actin was used to normalize the protein loading. (TIF) [file pone.0081269.s001.tif]

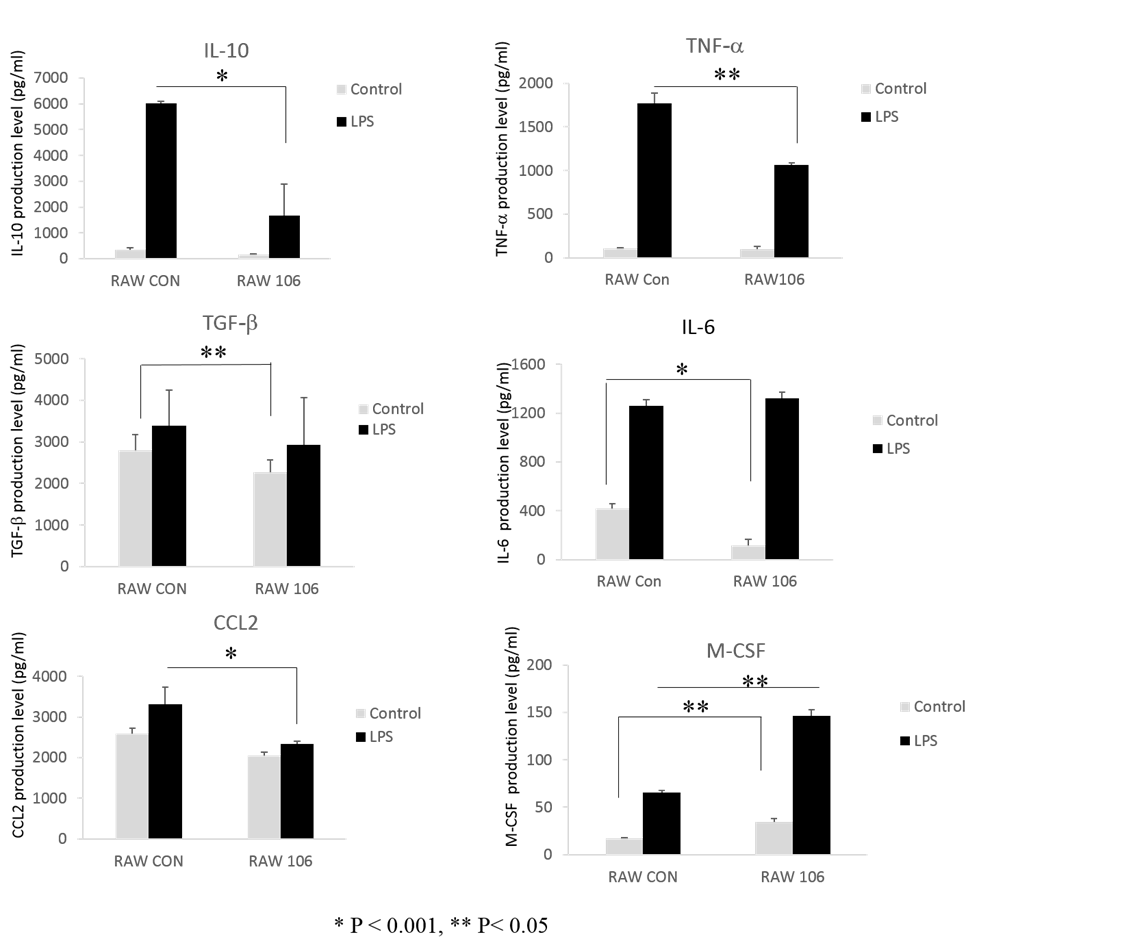

Supplement: Figure S2 — RNase L regulates the expression of cytokines and chemokines in macrophages. RNase L knocking down (Clone 106) and wild type Raw264.7 cells were treated with 1 µg/ml of LPS for 14 h. The secretory level of certain cytokines and chemokines in the media was measured by using an ELISA kit for each of the analyzers. Experiments were performed two times in triplicates. Data are presented as mean ±SD. *p<0.001, **p<0.05. (TIF) [file pone.0081269.s002.tif]
